# Nanoliposomal Irinotecan in Combination With 5‐Fluorouracil and Leucovorin for Advanced Head and Neck and Esophageal Squamous Cell Carcinoma After Prior Platinum‐Based Chemotherapy or Chemoradiotherapy: A Multicenter Phase II Trial

**DOI:** 10.1002/cam4.71307

**Published:** 2025-10-21

**Authors:** Muh‐Hwa Yang, Shang‐Yin Wu, I‐Wei Ho, Nai‐Jung Chiang, Chin‐Fu Hsaio, Chen‐Yuan Lin, Ming‐Yu Lien, Peter Mu‐Hsin Chang, Jia‐Hong Chen, Ching‐Yun Hsieh, Ruey‐Long Hong, Chao‐Tung Lee, Li‐Tzong Chen, Tsang‐Wu Liu, Chang‐Fang Chiu, Li‐Yuan Bai

**Affiliations:** ^1^ Department of Oncology Taipei Veterans General Hospital Taipei Taiwan; ^2^ Institute of Clinical Medicine National Yang Ming Chiao Tung University Taipei Taiwan; ^3^ Department of Oncology National Cheng Kung University Hospital, College of Medicine, National Cheng Kung University Tainan Taiwan; ^4^ School of Medicine, College of Medicine National Yang Ming Chiao Tung University Taipei Taiwan; ^5^ Institute of Population Health Sciences National Health Research Institutes Zhunan Taiwan; ^6^ Division of Hematology and Oncology, Department of Internal Medicine China Medical University Hospital Taichung Taiwan; ^7^ School of Pharmacy China Medical University Taichung Taiwan; ^8^ College of Medicine, School of Medicine China Medical University Taichung Taiwan; ^9^ Institute of Biopharmaceutical Sciences National Yang Ming Chiao Tung University Taipei Taiwan; ^10^ Division of Hematology and Oncology, Department of Internal Medicine Tri‐Service General Hospital Taipei Taiwan; ^11^ Department of Oncology National Taiwan University Taipei Taiwan; ^12^ National Institute of Cancer Research National Health Research Institutes Tainan Taiwan; ^13^ Department of Internal Medicine Kaohsiung Medical University Hospital, College of Medicine, Kaohsiung Medical University Kaohsiung Taiwan; ^14^ Center of Cancer Research, Kaohsiung Medical University Kaohsiung Taiwan; ^15^ Cancer Center, China Medical University Hospital Taichung Taiwan

**Keywords:** esophageal cancer, head and neck cancer, nanoliposomal irinotecan, platinum, squamous cell carcinoma

## Abstract

**Purpose:**

To evaluate the efficacy and safety of nanoliposomal irinotecan (nal‐IRI) plus 5‐fluorouracil (5‐FU) and leucovorin (LV) in patients with platinum‐refractory or intolerant head and neck squamous cell carcinoma (HNSCC) and esophageal squamous cell carcinoma (ESCC).

**Methods:**

In this multicenter, phase 2 study (NCT03712397), patients with advanced HNSCC (*n* = 43) or ESCC (*n* = 16) who had failed or were intolerant to platinum‐based chemotherapy received biweekly nal‐IRI 80 mg/m^2^ (equivalent to 70 mg/m^2^ of irinotecan base), LV 400 mg/m^2^, and 5‐FU 2400 mg/m^2^ until progression or unacceptable toxicity. The primary endpoint was the objective response rate (ORR).

**Results:**

In the intent‐to‐treat analysis, the ORR and disease control rate were 8.5% and 59.3% for the entire group. In the HNSCC subgroup, ORR and disease control rate were 11.6% and 65.1%, with a median progression‐free survival (PFS) of 2.7 months and an overall survival (OS) of 8.1 months. By contrast, no objective responses were observed in ESCC (ORR 0%, disease control rate 43.8%, median OS 4.2 months). The most common grade 3/4 toxicities were lymphopenia (50.8%), neutropenia (42.4%), leukopenia (33.9%), anemia (28.8%), and anorexia (8.5%).

**Conclusions:**

Nal‐IRI/5‐FU/LV demonstrates modest activity with acceptable safety profiles in patients with platinum‐refractory or intolerable advanced HNSCC. The exploratory findings warrant confirmation in larger, randomized studies.

**Trial Registration:**

ClinicalTrials.gov: NCT03712397

## 1 Introduction

Head and neck squamous cell carcinoma (HNSCC) is a critical health issue with over 600,000 new cases each year globally [1]. First‐line treatments for patients with recurrent or metastatic HNSCC typically consist of platinum‐based regimens before the introduction of pembrolizumab; however, median progression‐free survival (PFS) time is only 2.4–5.6 months [2, 3, 4]. Although there is no evidence of survival benefit demonstrated in randomized clinical trials, taxane, either as monotherapy or in combination with cetuximab and/or methotrexate, is a common practice for patients with disease progression after platinum‐based therapy [5, 6]. Patients with this condition have a very dismal prognosis, with a median overall survival (OS) of approximately 6 months [7, 8, 9].

Immune checkpoint inhibitors provide new prospects as second‐line treatments for platinum‐refractory HNSCC. Nivolumab and pembrolizumab have demonstrated significant efficacy in patients with platinum‐refractory HNSCC, achieving median OS of 7.5–8.4 months compared to 5.1–6.9 months in the control group [7, 8]. Moreover, the effectiveness of these treatments is limited to patients with PD‐L1 expression, which accounts for 80% of the patients in the KEYNOTE‐040 trial and 70% of the patients in the CheckMate 141 study [7, 8]. Therefore, there is an unmet need for patients with low PD‐L1 expression who are unsuitable for treatment with PD‐1 inhibitors after platinum failure.

Similarly, esophageal squamous cell carcinoma (ESCC) continues to be a major contributor to cancer mortality, particularly in Asia, Africa, and South America [1, 10]. The primary management for patients with metastatic or unresectable locally advanced ESCC is combination chemotherapy or chemoradiation with platinum and fluoropyrimidine, with or without checkpoint inhibitors [11]. The effectiveness of checkpoint inhibitors for disease progression after platinum treatment was limited to patients who were PD‐L1 positive in the trials. Notably, no significant survival benefit was observed for patients with PD‐L1 expression levels < 1% (50% of the study cohort) in the ATTRACTION‐3 study or for patients with a combined positive score (CPS) < 10 (65% of the study cohort) in the KEYNOTE‐181 trial [12, 13]. For individuals ineligible for immunotherapy following refractory platinum‐based treatment, monotherapies with taxanes or irinotecan with limited efficacy are recommended as subsequent treatment options [11].

Irinotecan, a topoisomerase I inhibitor, shows moderate activity in patients with HNSCC and ESCC. The combination of irinotecan and cisplatin has exhibited a response rate of 35% but with substantial toxicity in the first‐line treatment of patients with HNSCC [14]. In addition, irinotecan is advocated as a treatment option after the failure of platinum‐based regimens in patients with ESCC. Single‐agent irinotecan resulted in a response rate, median PFS, and median OS of 15%, 2 months, and 5 months, respectively, for cisplatin‐resistant ESCC in a phase II trial [15]. Combination therapy with irinotecan and fluoropyrimidine yielded a response rate, median PFS, and median OS of 17%–29%, 2.0–3.7 months, and 5.0–6.4 months, respectively [16, 17].

Nanoliposomal irinotecan (nal‐IRI, PEP02, MM‐398, Onivyde), encapsulating irinotecan within a nanoliposome, offers a novel approach for drug delivery and efficacy enhancement, with the goal of optimizing therapeutic outcomes and minimizing adverse effects [18]. In a phase I clinical trial, nal‐IRI monotherapy achieved a 21% response rate, accompanied by a manageable toxicity profile, in patients with advanced ESCC who were refractory or intolerant to previous chemotherapy [19]. Combination chemotherapy with nal‐IRI and 5‐fluorouracil (5‐FU) has been approved for patients with metastatic pancreatic ductal adenocarcinoma previously treated with gemcitabine‐based therapy [20]. The efficacy of nal‐IRI as a first‐line treatment for patients with pancreatic cancer was later successfully demonstrated in the NAPOLI 3 trial [21]. However, the effect of nal‐IRI/5‐FU/leucovorin (LV) in patients with HNSCC and ESCC after platinum‐based therapy remains unknown.

Considering the limited choice and unsatisfactory efficacy of current treatments for HNSCC and ESCC patients with disease progression after platinum‐based chemotherapy or chemoradiation therapy, moderate activity of irinotecan in HNSCC and ESCC, and improved delivery of irinotecan by nanoliposome, we conducted a prospective study to evaluate the efficacy and safety profiles of nal‐IRI/5‐FU/LV in patients with HNSCC and ESCC after platinum treatment in Taiwan.

## 2 Patients and Methods

### 2.1 Study Design

T2317 is a phase 2, multicenter, open‐label, single‐arm clinical study assessing the efficacy and safety of nal‐IRI/5‐FU/LV in patients with platinum refractory or intolerance advanced HNSCC and ESCC. Platinum refractoriness was defined as documented disease progression during or within 6 months after prior platinum‐based chemotherapy or concurrent chemoradiotherapy. Platinum intolerance was defined as intolerance to at least 6 weeks of platinum‐based systemic chemotherapy, or intolerance to at least 3 weeks of platinum‐based chemoradiation. Eligible patients must fulfill the following main criteria: adults (≥ 20 years) with histologically confirmed advanced, unresectable, locally advanced, recurrent, or metastatic HNSCC or ESCC; ineligible for surgical or radiation interventions; an Eastern Cooperative Oncology Group (ECOG) Performance Status (PS) score of 0 or 1; measurable disease by Response Evaluation Criteria in Solid Tumors v1.1 (RECIST 1.1) criteria; no prior exposure to nal‐IRI or irinotecan; adequate hematologic, hepatic, and renal functions; understand and sign the informed consent. The trial was conducted at five hospitals coordinated by the Taiwan Cooperative Oncology Group, National Health Research Institute of Taiwan.

### 2.2 Treatment

Participants were administered the nal‐IRI/5‐FU/LV regimen every 2 weeks: nanoliposomal irinotecan 80 mg/m^2^ (equivalent to 70 mg/m^2^ of irinotecan base) intravenously for 90 min on day 1, leucovorin 400 mg/m^2^ intravenously for 30 min on day 1, and 5‐fluorouracil 2400 mg/m^2^ intravenously over 46 h on day 1. The dosage of each medication was adjusted based on toxicities according to the specified criteria. Treatment was continued until disease progression, unacceptable toxicity, patient withdrawal, or other treatment discontinuation criteria were met.

### 2.3 Efficacy and Safety Assessments

The primary outcome measure was to assess the overall response rate (ORR) of nal‐IRI/5FU/LV in patients with advanced HNSCC or ESCC. The secondary outcome measures included disease control rate (DCR), duration of response, PFS, time to tumor progression (TTP), 1‐year survival rate, OS, and safety profiles.

The ORR was evaluated every 2 months using RECIST 1.1 guidelines. The DCR was defined as an ORR + stable disease rate. The PFS was calculated from the date of registration to the date of disease progression or death from any cause. OS was calculated from the date of registration to the date of death or last follow‐up. Adverse events were recorded and categorized according to CTCAE v5.0 criteria.

### 2.4 Sample Size

The study hypothesis aims to improve the response rate from 5% in the historical control to 15% with nal‐IRI/5‐FU/LV, with a power of 0.8 and a one‐sided type I error rate (*α*) of 5%. Accordingly, an initial cohort of 10–12 patients, with 5–6 patients each in HNSCC and ESCC groups, will be enrolled, treated, and evaluated by the Data and Safety Monitoring Board (DSMB) to assess the feasibility of the study regimen within the protocol‐defined patient population. If the DSMB deems the protocol feasible, an additional 18–20 patients will be enrolled according to Simon's minimax two‐stage design, bringing the total to 30 patients in stage 1 (without restricting the number of patients per cancer type). If two or more responders are observed during stage 1, enrollment to stage 2 will proceed with 22 additional patients, culminating in a total of 52 participants. Should five or more patients demonstrate a treatment response by the end of stage 2, the combination regimen will be considered effective and warrant further investigation.

### 2.5 Statistical Methods

Efficacy parameters were analyzed based on intent‐to‐treat (ITT) and measurable populations. The ITT population comprised all recruited participants who provided informed consent, and the measurable population included participants who received at least two cycles of study treatment. Safety profile analyses are performed in the ITT population. Both PFS and OS were plotted using the Kaplan–Meier method. Univariable Cox proportional hazards models were first used to explore the association between each covariate and survival outcomes. Subsequently, a multivariable Cox model was constructed, including all candidate variables of interest to obtain fully adjusted hazard ratios and 95% confidence intervals (CIs). Statistical analysis was performed using Statistical Analysis System (SAS) software (version 9.4, SAS Institute Inc., Cary, NC, USA). Data are expressed as mean ± standard deviation. All statistical tests were two‐sided, and differences were considered statistically significant at a *p*‐value ≤ 0.05.

## 3 Results

### 3.1 Patients Characteristics

The baseline demographic and clinical characteristics of the ITT and measurable populations are summarized in Table 1. The ITT population comprised 43 patients with advanced HNSCC and 16 patients with ESCC. The median age was 55 years (range, 40–79) and the average age was 55.3 ± 8.6 years, with males predominant (91.5%). More patients had a PS score of 1 (64.4%) than patients had PS score 0. Although only 13.6% of the patients had metastatic lesions, more than half of the patients had stage IV disease (64.4%) at the time of enrollment. All patients were enrolled in the study because of platinum refractoriness. The median number of treatment cycles for nal‐IRI/5‐FU/LV was four (range, 1–24).

**TABLE 1:** Baseline demographic and clinical characteristics.

Characteristics	ITT population *n* (%)	Measurable population *n* (%)
Number of patients	59	52
Age		
Mean ± SD	55.3 ± 8.6	54.8 ± 8.8
Median (range), years	55 (40–79)	55 (40–79)
Sex		
Male	54 (91.5)	48 (92.3)
ECOG PS score		
0	21 (35.6)	20 (35.8)
1	38 (64.4)	32 (61.5)
Cell differentiation		
Well differentiated	7 (11.9)	7 (13.5)
Moderately differentiated	33 (55.9)	30 (57.7)
Poorly differentiated	11 (18.6)	8 (15.4)
Undifferentiated	1 (1.7)	1 (1.9)
Other	2 (3.4)	2 (3.8)
Unknown	5 (8.5)	4 (7.7)
Site of the cancer		
Head & Neck	43 (72.9)	39 (75.0)
Esophagus	16 (27.1)	13 (25.0)
T stage		
T1	7 (11.9)	5 (9.6)
T2	7 (11.9)	7 (13.5)
T3	17 (28.8)	14 (26.9)
T4	28 (47.5)	26 (50.0)
N stage		
N0	15 (25.4)	12 (23.1)
N1	12 (20.3)	11 (21.2)
N2	20 (33.9)	18 (34.6)
N3	12 (20.3)	11 (21.2)
M stage		
M0	51 (86.4)	45 (86.5)
M1	8 (13.6)	7 (13.5)
Stage		
I	5 (8.5)	3 (5.8)
II	1 (1.7)	1 (1.9)
III	15 (25.4)	13 (25.0)
IV	38 (64.4)	35 (67.3)
Patient population		
Platinum refractoriness	59 (100.0)	52 (100.0)
Platinum intolerance	0 (0.0)	0 (0.0)

Abbreviations: ECOG PS, Eastern Cooperative Oncology Group performance status; ITT, intent‐to‐treat; SD, standard deviation.

### 3.2 Treatment Efficacy

The efficacy outcomes in the ITT population are summarized in Table 2. The ORR and DCR were 8.5% and 59.3%, respectively, for all participants. The median duration of response was 135.5 (range, 108–262) days. Specifically, the ORR and DCR were 11.6% and 65.1%, and 0% and 43.8%, respectively, for patients with HNSCC and ESCC.

**TABLE 2:** Treatment efficacy in intent‐to‐treat population.

	Head & neck *n* (%)	Esophagus *n* (%)	Overall *n* (%)
Number of patients	43	16	59
Evaluable	39 (90.7)	14 (87.5)	53 (89.8)
Complete response	1 (2.3)	0 (0.0)	1 (1.7)
Partial response	4 (9.3)	0 (0.0)	4 (6.8)
Stable disease	23 (53.5)	7 (43.8)	30 (50.8)
Progressive disease	11 (25.6)	7 (43.8)	18 (30.5)
Not evaluable	4 (9.3)	2 (12.5)	6 (10.2)
Duration of response			
Mean(days) ± SD	161.3 ± 69.3	^a^	161.3 ± 69.3
Median	137.5	^a^	137.5
Min/Max	108/262	^a^	108/262

Abbreviation: SD, standard deviation.

^a^ No responder.

**FIGURE 1:**
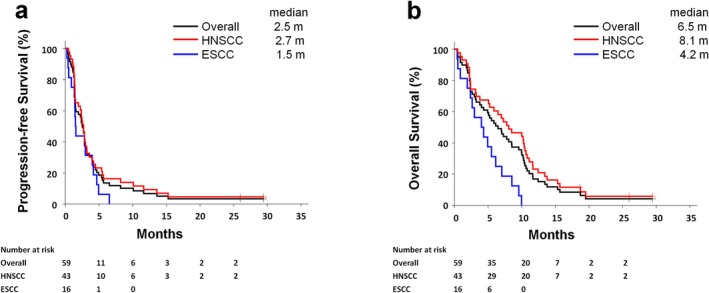
Kaplan–Meier curve for survival in intent‐to‐treat population. (a) Progression‐free survival and (b) overall survival according to cancer types.

Survival analysis in the ITT population was summarized in Table 3. The median PFS, median OS, and 1‐year survival rate for OS were 2.5 (95% CI, 1.5–3.0) months, 6.5 (95% CI, 4.0–8.5) months, and 17%, respectively, for the whole population (Figure 1). Patients with HNSCC had a longer PFS and OS than patients with ESCC. For patients with HNSCC, the median PFS, median OS, and 1‐year survival rate for OS were 2.7 (95% CI, 1.5–3.2) months, 8.1 (95% CI, 5.1–10.5) months, and 23%, respectively (Figure 1). Multivariable Cox regression analysis found that performance status was the most important factor for overall survival (Table S1).

**TABLE 3:** Survival result in ITT analysis.

	Esophagus	Head & neck	Overall
Number of patients	16	43	59
Survival analysis (months)			
mPFS (95% CI)	1.5 (0.9–4.0)	2.7 (1.5–3.2)	2.5 (1.5–3.0)
mOS (95% CI)	4.2 (1.6–6.1)	8.1 (5.1–10.5)	6.5 (4.0–8.5)
1‐years survival rate (95% CI)	0	23 (12–37)	17 (9–28)

Abbreviations: CI, confidence interval; ITT, intent‐to‐treat; mOS, median overall survival; mPFS, median progression‐free survival; NA, not available.

The efficacy outcomes and survival analysis for the measurable population are summarized in Supporting Information (Tables S2 and S3, Figure S1).

### 3.3 Adverse Events

Treatment emergent adverse events with an overall incidence of ≥ 5% are shown in Table 4. Hematological toxicities were the main toxicities. Grade 3 or higher toxicities included lymphopenia (50.8%), neutropenia (42.4%), leukopenia (33.9%), anemia (28.8%), anorexia (8.5%), nausea (6.8%), diarrhea (5.1%), fatigue (5.1%), and thrombocytopenia (5.1%). No treatment‐related death occurred in the study.

**TABLE 4:** Treatment emergent adverse events with overall incidence ≥ 5% (*N* = 59).

Adverse effects	Grade 1	Grade 2	Grade 3	Grade 4	Grade 5
Hematologic adverse events					
Anemia	7 (11.9%)	26 (44.1%)	17 (28.8%)	0 (0.0%)	0 (0.0%)
White blood cell decreased	7 (11.9%)	14 (23.7%)	17 (28.8%)	3 (5.1%)	0 (0.0%)
Lymphocyte count decreased	1 (1.7%)	8 (13.6%)	19 (32.2%)	11 (18.6%)	0 (0.0%)
Neutrophil count decreased	3 (5.1%)	9 (15.3%)	21 (35.6%)	4 (6.8%)	0 (0.0%)
Platelet count decreased	10 (16.9%)	1 (1.7%)	2 (3.4%)	1 (1.7%)	0 (0.0%)
Non‐hematologic adverse events					
Fatigue	27 (45.8%)	16 (27.1%)	3 (5.1%)	0 (0.0%)	0 (0.0%)
Anorexia	24 (40.7%)	8 (13.6%)	5 (8.5%)	0 (0.0%)	0 (0.0%)
Diarrhea	18 (30.5%)	9 (15.3%)	3 (5.1%)	0 (0.0%)	0 (0.0%)
Nausea	17 (28.8%)	9 (15.3%)	4 (6.8%)	0 (0.0%)	0 (0.0%)
Vomiting	16 (27.1%)	7 (11.9%)	2 (3.4%)	0 (0.0%)	0 (0.0%)
Weight Loss	15 (25.4%)	8 (13.6%)	0 (0.0%)	0 (0.0%)	0 (0.0%)
Hypoalbuminemia	11 (18.6%)	5 (8.5%)	0 (0.0%)	0 (0.0%)	0 (0.0%)
Oral mucositis	9 (15.3%)	5 (8.5%)	2 (3.4%)	0 (0.0%)	0 (0.0%)
Fever	6 (10.2%)	5 (8.5%)	0 (0.0%)	0 (0.0%)	0 (0.0%)
Hyponatremia	7 (11.9%)	3 (5.1%)	0 (0.0%)	1 (1.7%)	0 (0.0%)
Constipation	8 (13.6%)	0 (0.0%)	0 (0.0%)	0 (0.0%)	0 (0.0%)
ALK‐P increased	3 (5.1%)	1 (1.7%)	0 (0.0%)	0 (0.0%)	0 (0.0%)
Hypophosphatemia	4 (6.8%)	0 (0.0%)	0 (0.0%)	0 (0.0%)	0 (0.0%)
Creatinine increased	2 (3.4%)	1 (1.7%)	0 (0.0%)	0 (0.0%)	0 (0.0%)
γGT increased	2 (3.4%)	0 (0.0%)	1 (1.7%)	0 (0.0%)	0 (0.0%)

Abbreviations: γGT, γ‐glutamyl transferase; ALK‐P, alkaline phosphatase.

## 4 Discussion

Our study represents the first investigation of nal‐IRI/5‐FU/LV as a second‐line treatment for patients with HNSCC and ESCC after failure of platinum‐based therapies. Given the limited treatment options and the unsatisfactory efficacy of salvage regimens, there remains a significant unmet clinical need in the groups of patients. In this context, nal‐IRI/5‐FU/LV demonstrated a modest efficacy in patients with HNSCC, achieving an ORR of 11.6%, a DCR of 65.1%, a median PFS of 2.7 months, and a median OS of 8.1 months.

While our study enrolled patients with HNSCC and ESCC, we acknowledge that these are biologically and clinically distinct malignancies with different etiologies, molecular characteristics, and clinical courses. Our rationale for targeting HNSCC and ESCC was based on their shared platinum‐refractory scenario and the same unmet medical need for effective second‐line treatments after failure of platinum‐based chemotherapy or chemoradiation. Indeed, while there was modest activity of nal‐IRI/5‐FU/LV in HNSCC, the response of ESCC was disappointing.

For HNSCC who fail platinum‐based chemotherapy or chemoradiation, no specific treatment is recommended despite the availability of various agents, including paclitaxel, docetaxel, methotrexate, afatinib, cetuximab, and capecitabine [5, 22]. The LUX‐Head & Neck 1 phase III study compared the efficacy of oral afatinib with that of methotrexate in patients with HNSCC progressing during or after platinum‐based therapy [9]. Afatinib prolonged the primary endpoint of PFS (2.6 vs. 1.7 months) compared to methotrexate. The response rate and OS were 10% and 6%, and 6.8 and 6.0 months for afatinib and methotrexate treatment, respectively [9]. Taxane and irinotecan are commonly used chemotherapeutic regimens for ESCC patients refractory to platinum‐based therapy [11], with a median OS ranging from 6.2 to 8.4 months [12, 13, 23, 24].

Currently, immune checkpoint inhibitors are superior to alternative systemic therapies for patients with HNSCC and ESCC who have progressed on or after platinum‐based therapies, according to phase 3 clinical trials [7, 8, 12, 13, 23, 24]. However, a major concern under these circumstances is the low activity of checkpoint inhibitors in patients with low PD‐L1 expression. For patients with HNSCC treated with pembrolizumab, no OS benefit was noted in patients with a PD‐L1 CPS < 1 compared with the standard‐of‐care group. The median OS was 6.3 and 7.0 months, respectively [7]. As well, nivolumab did not confer survival prolongation in patients with a PD‐L1 expression < 1% compared to the standard‐of‐care group, with median OS of 5.7 and 5.8 months, respectively [8]. Importantly, the population of non‐beneficiary encompassed 20%–30% of the entire study population [7, 8]. Because PD‐L1 expression was not assessed in our study population, we cannot determine whether the observed efficacy of nal‐IRI/5‐FU/LV differs between CPS < 1 and CPS ≥ 1 subgroups for HNSCC patients. Future randomized studies should stratify patients by CPS status, with particular emphasis on the CPS < 1 subgroup, to determine whether nal‐IRI/5‐FU/LV may represent an effective alternative for patients with limited benefit from checkpoint inhibitors.

Nal‐IRI, with the goal of optimizing therapeutic outcomes and minimizing adverse effects through a novel drug delivery approach, resulted in a 21% response rate in patients with advanced ESCC that was refractory or intolerant to previous chemotherapy, accompanied by a manageable toxicity profile in a phase I study [19]. The superiority of the combination of nal‐IRI and 5‐FU over nal‐IRI monotherapy has been shown in patients with metastatic pancreatic ductal adenocarcinoma previously treated with gemcitabine‐based therapy [20]. To date, no studies have reported nal‐IRI/5‐FU/LV in patients with HNSCC and ESCC, either after platinum‐based or treatment‐naïve therapy. Currently, there is an ongoing phase II trial (OESIRI) to test nal‐IRI plus 5‐FU versus paclitaxel as second‐line therapy in metastatic ESCC [25].

The safety profile of nal‐IRI/5‐FU/LV observed in our study is consistent with previous reports in other solid tumors [20, 26]. Only one patient (1.7%) required more than two dose reductions, and no treatment discontinuations occurred due to adverse events. Notably, a higher proportion of patients in our study experienced grade 3 or higher anemia compared to those in the NAPOLI‐1 and NIFTY studies, with an incidence rate of 28% in our study versus 9% in both the NAPOLI‐1 and NIFTY studies [20, 26]. One possible explanation is the prior administration of platinum in the current study compared with that of gemcitabine in the NAPOLI‐1 study. In contrast, the incidence of grade 3/4 diarrhea was higher in the NAPOLI‐1 trial (13% vs. 5.1%), which involved patients with pancreatic cancer [20].

Several limitations of this study merit consideration. First, this was a phase 2 trial with a limited number of patients, no control arm, and inclusion of two biologically distinct disease entities. Second, UGT1A1 genotyping was not mandatory for enrollment. Although a lower starting dose of nal‐IRI is recommended for patients homozygous for UGT1A1*28, pre‐screening was not performed due to the low prevalence of this genotype in Southeast Asia [27]. Nonetheless, the absence of UGT1A1 testing represents a limitation, as pharmacogenomic variability could have influenced treatment‐related toxicity, and future studies should incorporate genotyping into trial design, particularly in East Asian populations. Third, no biomarker analyses such as PD‐L1 expression, human papillomavirus status, tumor mutational burden, or cancer‐associated fibroblast‐derived miRNAs were assessed [28]. The lack of biomarker correlation limits our ability to identify subgroups that might derive greater benefit from nal‐IRI/5‐FU/LV therapy. Taken together, these limitations indicate that our findings are exploratory and need to be validated in larger, randomized trials.

## 5 Conclusions

In conclusion, the findings of the current phase 2 study imply that the nal‐IRI/5‐FU/LV regimen may represent a potential treatment option for patients with HNSCC previously treated with platinum‐based regimens. This regimen resulted in manageable and predominantly reversible adverse events. However, given the modest efficacy and the limitations of a small, single‐arm design, the results should be regarded as exploratory and hypothesis‐generating. Confirmation in larger, randomized trials is required before clinical adoption can be considered.

## Author Contributions

**Muh‐Hwa Yang:** data curation, investigation, formal analysis, writing – review and editing. **Shang‐Yin Wu:** data curation, investigation, formal analysis, writing – review and editing. **I‐Wei Ho:** formal analysis, writing – original draft, writing – review and editing. **Nai‐Jung Chiang:** data curation, investigation, project administration, writing – review and editing. **Chin‐Fu Hsaio:** formal analysis, methodology, software, validation, writing – review and editing. **Chen‐Yuan Lin:** investigation, data curation, writing – review and editing. **Ming‐Yu Lien:** investigation, data curation, writing – review and editing. **Peter Mu‐Hsin Chang:** data curation, investigation, writing – review and editing. **Jia‐Hong Chen:** data curation, investigation, writing – review and editing. **Ching‐Yun Hsieh:** data curation, investigation, writing – review and editing. **Ruey‐Long Hong:** data curation, investigation, writing – review and editing. **Chao‐Tung Lee:** software, validation, methodology, formal analysis, writing – review and editing. **Li‐Tzong Chen:** conceptualization, funding acquisition, resources, project administration, supervision, writing – review and editing. **Tsang‐Wu Liu:** conceptualization, funding acquisition, project administration, supervision, resources, writing – review and editing. **Chang‐Fang Chiu:** conceptualization, funding acquisition, project administration, resources, writing – review and editing. **Li‐Yuan Bai:** conceptualization, formal analysis, writing – original draft, writing – review and editing.

## Ethics Statement

This study was performed in line with the principles of the Declaration of Helsinki. The study protocol was approved by the Institutional Review Board of each participating hospital: National Health Research Institutes Research Ethics Committee (EC1080201‐E), National Taiwan University Hospital Research Ethics Committee (NTUH 01901025MS), Institutional Review Board, Tri‐Service General Hospital, National Defense Medical Center (TSGH‐108‐01‐002), Institutional Review Board of Taipei Veterans General Hospital (VGH‐TPE 018‐12‐008C), China Medical University Hospital Research Ethics Committee (CMUH107‐REC2‐137), and National Cheng Kung University Hospital Institutional Review Board (A‐BR107‐067).

## Conflicts of Interest

The authors declare no conflicts of interest.

## Supporting information


**Figure S1:** Kaplan–Meier curve for survival in measurable population. (a) Progression‐free survival and (b) overall survival according to cancer types.

**Table S1:** Cox regression of overall survival in intent‐to‐treat population.
**Table S2:** Treatment efficacy in measurable population.
**Table S3:** Survival results in measurable population.

## Data Availability

All relevant data is within the manuscript and Supporting Information.
