# Supplementary figures and images for: Nanoliposomal Irinotecan in Combination With 5‐Fluorouracil and Leucovorin for Advanced Head and Neck and Esophageal Squamous Cell Carcinoma After Prior Platinum‐Based Chemotherapy or Chemoradiotherapy: A Multicenter Phase II Trial
Source: Cancer Med. 2025 Oct 21;14(20):e71307. doi: 10.1002/cam4.71307 (PMC12538807; doi:10.1002/cam4.71307)

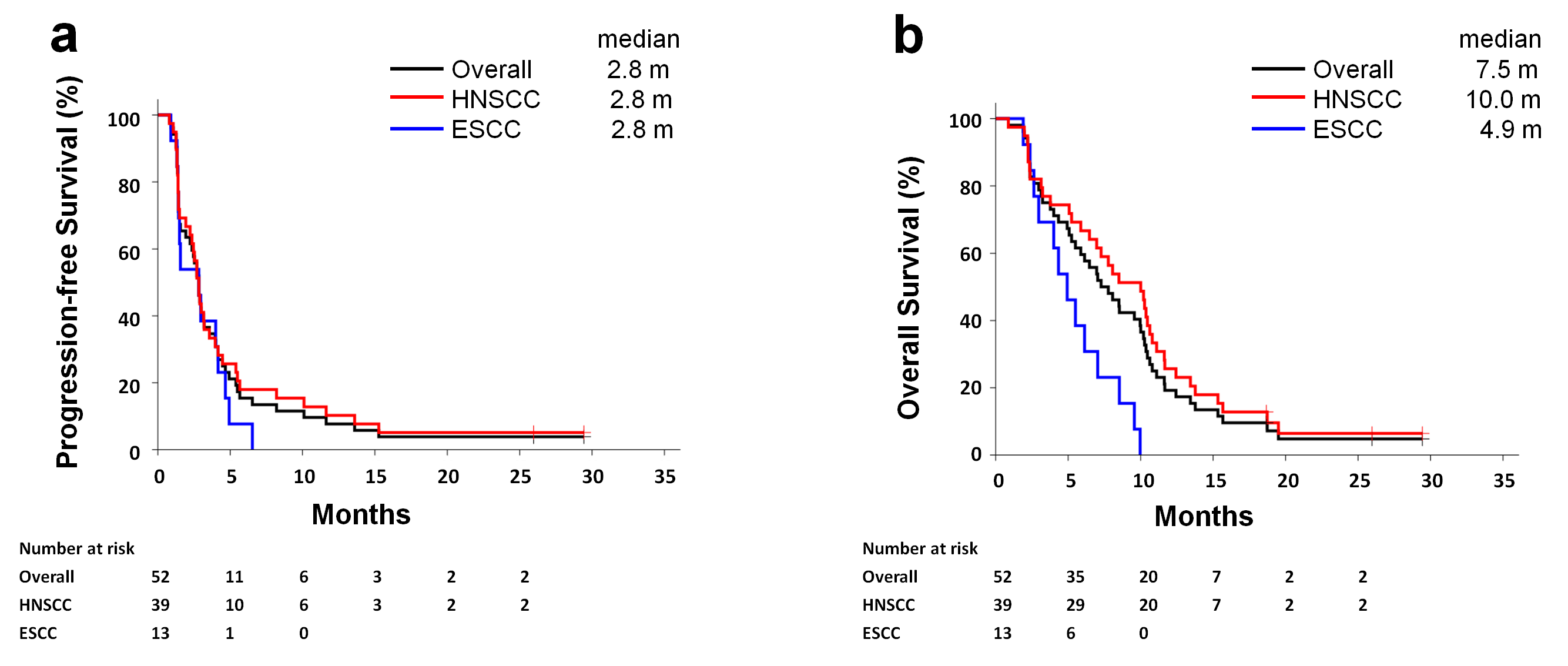

Supplement: Supplementary file 1 — Figure S1: Kaplan–Meier curve for survival in measurable population. (a) Progression‐free survival and (b) overall survival according to cancer types. [file CAM4-14-e71307-s001.tif]
