# Supplementary material for: Nanoliposomal Irinotecan in Combination With 5‐Fluorouracil and Leucovorin for Advanced Head and Neck and Esophageal Squamous Cell Carcinoma After Prior Platinum‐Based Chemotherapy or Chemoradiotherapy: A Multicenter Phase II Trial
Source: Cancer Med. 2025 Oct 21;14(20):e71307. doi: 10.1002/cam4.71307 (PMC12538807; doi:10.1002/cam4.71307)
Supplement: Supplementary file 2 — Table S1: Cox regression of overall survival in intent‐to‐treat population. Table S2: Treatment efficacy in measurable population. Table S3: Survival results in measurable population. [file CAM4-14-e71307-s002.docx]

# TABLE S1. Cox regression of overall survival in intent-to-treat population ^22^

|  |  | **Simple Regression** | | | | **Multiple Regression** | | |
| --- | --- | --- | --- | --- | --- | --- | --- | --- |
|  | **Event/n** | **HR** | **95% CI** | **P-value** | **HR** | | **95% CI** | **P-value** |
| Sex (male vs female) | 51/54 vs 5/5 | 0.57 | (0.23, 1.45) | 0.24 | 0.38 | | (0.13, 1.15) | 0.09 |
| Age (≥50 vs < 50 years) | 39/42 vs 17/17 | 0.67 | (0.38, 1.20) | 0.18 | 1.05 | | (0.53, 2.05) | 0.90 |
| Performance status (1 vs 0) | 38/38 vs 18/21 | 2.39 | (1.31, 4.35) | <0.01 | 3.43 | | (1.56, 7.56) | <0.01 |
| T stage (4 vs 1/2/3) | 27/28 vs 29/31 | 0.78 | (0.46, 1.34) | 0.37 | 0.52 | | (0.25, 1.07) | 0.08 |
| N stage (2/3 vs 0/1) | 31/32 vs 25/27 | 1.29 | (0.75, 2.22) | 0.36 | 1.50 | | (0.75, 2.99) | 0.25 |
| M stage (1 vs 0) | 8/8 vs 48/51 | 1.29 | (0.60, 2.75) | 0.52 | 0.89 | | (0.38, 2.10) | 0.79 |
| Stage (4 vs 1/2/3) | 37/38 vs 19/21 | 0.92 | (0.52, 1.63) | 0.77 | 1.00 | | (0.41, 2.42) | 1.00 |
| Cancer site (esophagus vs head & neck) | 16/16 vs 40/43 | 2.85 | (1.49, 5.46) | <0.01 | 1.61 | | (0.67, 3.87) | 0.29 |

Abbreviations: CI, confidence interval; HR, hazard ratio

TABLE S2. Treatment efficacy in measurable population

|  | **Head & Neck**  **n (%)** | **Esophagus**  **n (%)** | **Overall**  **n (%)** |
| --- | --- | --- | --- |
| Number of patients | 39 | 13 | 52 |
| Complete remission | 1 (2.6) | 0 (0.0) | 1 (1.9) |
| Partial response | 4 (10.3) | 0 (0.0) | 4 (7.7) |
| Stable disease | 23 (59.0) | 7 (53.8) | 30 (57.7) |
| Progress disease | 11 (28.2) | 6 (46.2) | 17 (32.7) |
| Duration of Response |  |  |  |
| Mean(days) ± SD | 161.3 ±69.3 | * | 161.3 ±69.3 |
| Median | 137.5 | * | 137.5 |
| Min/Max | 108/262 | * | 108/262 |

Abbreviations: SD, standard deviation

* No responder

TABLE S3. Survival results in measurable population

|  | **Esophagus** | **Head & Neck** | **Overall** |
| --- | --- | --- | --- |
| Number of patients | 13 | 39 | 52 |
| Survival analysis (months) |  |  |  |
| mPFS (95% CI) | 2.8 (1.4-4.2) | 2.8 (1.9-3.6) | 2.8 (1.9-3.2) |
| mOS (95% CI) | 4.9 (2.6-7.0) | 10.0 (5.9-10.8) | 7.5 (5.2-10.0) |
| 1-years survival rate (95% CI) | NA | 23 (11-37) | 19 (10-31) |

Abbreviations: CI, confidence interval; mOS, median overall survival; mPFS, median progression-free survival; NA, not available
